# Lessons learned from virtual arts-based resilience workshops for military healthcare providers: a community case study

**DOI:** 10.3389/fpsyg.2026.1851912

**Published:** 2026-07-22

**Authors:** Nathaniel McLaughlan, Rebecca Vaudreuil, Elizabeth K. Freeman, Hannah Jacobson Blumenfeld, Mallory Van Fossen, Kimberly Walter

**Affiliations:** 1 Henry M. Jackson Foundation for the Advancement of Military Medicine, Inc., Bethesda, MD, United States; 2 National Intrepid Center of Excellence at Walter Reed National Military Medical Center, Bethesda, MD, United States; 3 Independent Researcher, Santa Barbara, CA, United States

**Keywords:** community engagement, creative arts, creative arts therapies, military healthcare system, pandemic (COVID-19), provider support, resilience-building, telehealth

## Abstract

Healthcare providers contended with unique work challenges during the height of the COVID-19 pandemic by adapting to rapidly changing circumstances with limited knowledge and resources. One military treatment facility responded to these challenges by leveraging available technology to implement Operation Oasis, a communally developed and peer-led virtual resiliency program for healthcare providers. This paper aims to: (1) discuss the design and implementation of Operation Oasis, and (2) conduct a thematic analysis of 404 participant chat messages that participants voluntarily and anonymously provided across 2 years of a weekly Operation Oasis music-based offering, Human Jukebox. Key findings of the thematic analysis included that the “Music Appreciation” theme was in 43% of coded messages, making it the most common theme; “Positivity,” “Gratitude,” “Reminiscence/Nostalgia/Relating to Music,” and “Social Connectedness” comprised the remaining top five themes. Participants’ comments suggested they had experiences of positive emotions, personal reflection, and interpersonal connection, all of which are recognized as contributors to resilience and well-being. This paper offers workshop facilitators’ reflections and lessons learned on the process of creating and implementing Operation Oasis, as well as a discussion of the potential need and impact of supporting similar programs in the future.

## 1 Introduction

The COVID-19 pandemic shifted healthcare services on a global scale as professionals adapted their workflow and modified the way they delivered services (Bornheimer et al., 2022; Cotner et al., 2022). Telehealth platforms were adopted as necessary tools for continuing healthcare access during lockdown periods and have demonstrated benefits and challenges for patients and providers (Lai et al., 2020; Meese et al., 2022). Rapid and unpredictable changes in official telehealth guidance led to issues involving healthcare provision including missed appointments, patient wait lists, lack of telework policies, dissolution of work-life balance, and reduction of interactions between colleagues (Nair et al., 2021). Interruptions in provider workflow impacted patient well-being within mental health treatment environments as understaffing due to exposure, illness, or mandated quarantining efforts restricted the amount of time available per patient, which reduced access to and thoroughness of suicide risk assessments (Bornheimer et al., 2022). Front-line healthcare providers also described experiencing high levels of uncertainty around public health best practices, inconsistent leadership communication, and dissonance between broader pandemic guidelines and the needs of specific patients (Nelson et al., 2021). These conditions were associated with reported increases in provider stress, anxiety, depression, and burnout levels (Goss et al., 2022).

As providers became increasingly disconnected from their patients, communities, and one another, and adjusted to new parameters of telework, the impact of pandemic-related stressors warranted a response. There was a need for collaboration and organization among clinical, administrative, and technological resources to create support systems that bolstered provider resilience and improved familiarity with existing available technology for remote clinical practice (Rushton et al., 2022). This transition to utilizing remote workplace technologies has similar demands on employees across a myriad of professional contexts, and therefore the process has shown to be improved by similar implementations of institutional support. These factors include technology skills training, strong team communication, positive workplace relationships, and department leadership that embraced a technology-oriented workplace culture (Trenerry et al., 2021).

In addition to bolstering technology literacy, these support systems also offered socially oriented coping tools to address acute and chronic provider distress. Programming with beneficial outcomes incorporated psychoeducation in developing effective personal coping strategies that participants would use individually, while also conducting learning experiences within a social context. This approach demonstrated outcome measures of increased sense of resiliency and quality of life indicators, as well as decreased perception of isolation and use of avoidance-based coping strategies (Jubin et al., 2022). Furthermore, when workshops included experiential components focused on mindfulness and self-care skills, measures showed decreases in depression scales (Bechard et al., 2021) and increases in both resilience and empathy scales consistent with subjective self-reporting (Kim et al., 2022).

A noteworthy connection between some programs was the use of creative arts modalities to provide participants with expressive and sensory methods of both self-awareness and interactivity. Some virtual workshops invited participants to generate creative works that represent themselves and experience the creations of others. Participants could engage at their own level while balancing the risk-taking of sharing with the safety of witnessing. This combination resulted in an enhanced sense of social support, self-awareness, and internalized locus of control (Matto and Sullivan, 2021), as well as post-traumatic growth (Bechard et al., 2021). Other workshops were more focused on participants connecting with others through synchronous viewing of live performances and discussing their perspectives and responses. Analyzing the impact of such events highlighted predominant themes of interactivity, unity, resilience, identity, and emotional identification (Fraser et al., 2021). These approaches showed that using symbolic mediums for self-expression within a community beyond the self can enhance the potential benefits of engagement.

Underpinned by existing literature on resiliency building through arts-based and expressive social engagement, this paper focuses on the design and implementation of Operation Oasis, a virtual, peer-led provider resiliency program at a United States (U.S.) military treatment facility (MTF). Operation Oasis was created at the outset of the COVID-19 pandemic as a response to the need for provider support. To gain preliminary insights into participants’ experiences and the impact of one Operation Oasis offering, Human Jukebox (HJB), this paper includes an exploratory thematic analysis of chat messages from this music-based interactive workshop.

### 1.1 Creative arts therapies

Creative arts therapies (CATx) are healthcare professions that use arts-based processes in a clinical context to address physiological, psychological, and social well-being of individuals and communities (Shafir et al., 2020). Research suggests that CATx treatment may facilitate recovery from traumatic experiences through meaning-making (Berberian et al., 2019), positive reframing (Bradt et al., 2019), and externalization through image-making, symbolic expression, and the non-verbal-to-verbal shift that supports memory articulation (Smith, 2016). Further CATx treatment promotes the development of healthy independent coping mechanisms (Kaimal et al., 2019; Vaudreuil et al., 2020), anger and anxiety management through creative expression and improved self-regulation (Bronson et al., 2018), ability to experience hope and gratification (Berberian et al., 2019), and reduction of isolation through meaningful interactions (Vaudreuil et al., 2019; Walker et al., 2017).

Similar to many other healthcare disciplines, clinical facilitation of CATx services shifted to telehealth delivery during the COVID-19 lockdown periods (Clements-Cortés et al., 2023; Spooner et al., 2019; Vaudreuil et al., 2020). As the COVID-19 pandemic persisted as a public health concern, CATx practitioners also saw an increasing need for resources and support across a spectrum of engagement (e.g., clinical and non-clinical). Telehealth wellness and resilience workshops like Operation Oasis provided opportunities for individuals and communities to connect through collective engagement in creative processes.

### 1.2 Arts in Health

Arts in Health (AIH) refers to a diverse field composed of many creative subfields that recognizes the central role of the arts to promote health and wellbeing in institutional and community settings (National Organization for Arts in Health, 2017). Implementing the arts into diverse contexts is aimed at promoting, maintaining, or improving physical, mental, and social wellbeing (Fancourt and Finn, 2019). In hospital settings, activities such as participatory arts, arts at the bedside, visual exhibits, public performances, as well as arts-based training and education for healthcare staff fall within the scope of AIH programming.

A scoping review by Fancourt and Finn (2019) found that AIH programming demonstrated a wide variety of positive outcomes including affecting the social determinants of health, encouraging health-promoting behaviors, and supporting caregivers. Reduction in depression and anxiety symptoms (Stuckey and Nobel, 2010) and the enhancement of physical health through outcomes such as lowered blood pressure (Clow and Fredhoi, 2006) can be achieved through arts engagement. Furthermore, AIH programs can assist in pain management (Puetz et al., 2013), improve memory (Jiao et al., 2026), and help participants foster greater social connectedness while decreasing feelings of loneliness (Fancourt et al., 2021).

## 2 Context

Executing Operation Oasis required internal, external, and multi-agency collaboration from institutions including Walter Reed National Military Medical Center (Walter Reed) and the National Endowment for the Arts (NEA), Creative Forces®: NEA Military Healing Arts Network (CF). The NEA, in partnership with the Department of War (DoW) and Veterans Affairs, operates CF. The Walter Reed AIH Program provides the hospital community-at-large (e.g., patients, families, caregivers, staff, trainees, visitors) with arts engagement opportunities. These include CATx treatment, community arts programming, environmental arts, and bedside arts, each supporting different aspects of health (Walter Reed National Military Medical Center, 2019). CF positions creative arts therapists to work at military and veteran clinics across the U.S. and supports research efforts that aim to demonstrate the value and impact of the arts including clinical CATx treatment and community arts engagement (National Endowment for the Arts, 2025). These programs operate within the National Intrepid Center of Excellence (NICoE), an interdisciplinary clinic dedicated to the assessment and treatment of traumatic brain injury (TBI) and co-occurring psychological health challenges including post-traumatic stress disorder (PTSD) (Military Health System, 2024). CATx treatment is facilitated by art therapists, dance/movement therapists, and music therapists, with support from CF.

### 2.1 Creative Forces community programming

The relationship between clinical CATx and community-based arts programming is a critical component of the CF initiative and was foundational to developing Operation Oasis. CF community engagement efforts provide military-connected populations with continued arts opportunities that promote various health outcomes as shown in Table 1. These target outcomes were identified through monitoring, evaluation, and learning processes led by external subject matter experts through surveys, focus groups, and consultation with CF community engagement project leads alongside creative arts therapists and community arts facilitators.

**Table 1:** Creative Forces fact sheet (National Endowment for the Arts, 2025).

Outcome	Description
Empathy	Participants have a better understanding of themselves and others by creating or engaging with art.
Social Connectedness	Participants have supportive relationships in their life and a sense of belonging to a community
Resilience	Participants have developed coping tools to help them rebound from stress, unexpected events, or life’s challenges.
Independence and successful adjustment	Participants have an individual and shared sense of purpose and positive self-worth that supports difficult life transitions.

Taken together, these outcome targets corresponded to the design intentions of remotely accessible social environments that promoted adaptation to disruptive changes through engagement in meaningful social experiences and aimed to meet the unique needs of healthcare provider communities.

### 2.2 Operation Oasis formation

When the COVID-19 pandemic began to impact the U.S. in March 2020, most in-person clinical programming and research activities at Walter Reed were temporarily put on hold. In April 2020, team members from various departments collaborated to address the needs, challenges, and concerns imposed by COVID-19 pandemic regulations on staff. Because CF seeks to explore the relationship between clinical CATx treatment in healthcare and arts engagement in communities (National Endowment for the Arts, 2025), and the Walter Reed AIH Program aims to provide MTF stakeholders opportunities to engage with the arts to support health and well-being (Walter Reed National Military Medical Center, 2019), both were aptly positioned to collaboratively develop Operation Oasis.

Operation Oasis was a response to the “fear, anxiety, and frustration [that was] palpable every day in both patients and providers across the military treatment facility” (McGuire, M., Walter Reed AIH Program Founder, verbal communication, 03 May 2023). The initial vision for Operation Oasis was an in-person caregiver wellness program, but as the timeframe of physical distancing mandates continued to be extended, remained uncertain, and work status shifted between in-person, telework, and hybrid, the concept transitioned to virtual delivery.

The mission of Operation Oasis was to provide rest and respite for hospital staff, particularly those directly engaged in screening and caring for patients with COVID-19, by offering opportunities for creative and expressive engagements to support self-care and well-being (Walter Reed National Military Medical Center, 2019). A primary component of Operation Oasis was that it used a peer-support model and wellness-centered approach to facilitate workshops aimed at prevention through building community and resilience by connecting providers across different disciplines.

## 3 Key programmatic elements

Operation Oasis presented community programming within a clinical setting. This combination is inherently complex, especially in the context of heightened risk management, professional boundaries, and variance in definitions and expectations of clinical CATx treatment and community arts engagement. The process for creating Operation Oasis was built by leveraging the mission of the Walter Reed AIH Program. It was critical to have key stakeholders working together to support the sustainability of Operation Oasis. Design elements emerged from a learning curve of coordinated efforts to identify facilitators, promote a variety of programming, create and disseminate a weekly schedule of offerings to providers, and provide administrative support.

The final iteration of Operation Oasis yielded a balanced delegation of duties. Clinical staff were responsible for developing content that could be effectively transmitted virtually and the NICoE administrative staff were responsible for coordinating workshops, schedule distribution, and operating the approved digital platform (i.e., opening/closing the virtual meeting rooms, admitting participants). Both roles involved new and unfamiliar experiences, thus requiring flexibility and creativity of personnel. This collaborative process evolved as both administrative staff and providers learned more about the capabilities of the available and approved hardware and software technologies and gleaned which approaches were most effective. One such arts-based offering, HJB, is detailed below.

### 3.1 Human Jukebox (HJB)

Two board-certified music therapists affiliated with Creative Forces—each with more than 5 years of experience working in military healthcare settings—developed and co-facilitated the HJB workshop. HJB entailed 45-min, weekly live-streamed music-centered group workshops that incorporated elements of audience participation. The tools of interaction for the facilitators were the audio (microphones) and video (webcams), interactive computer software, musical instruments of choice (guitar, ukulele, percussion, voice), as well as live chat and pre-prepared on-screen text within the virtual meeting program. Participants could interact using their personal devices (computer or smartphone) by clicking on-screen poll prompts and contributing to the live chat feed that was viewable by facilitators and participants.

HJB aimed to cultivate a stress-reducing and morale-boosting atmosphere for healthcare providers seeking respite from work patterns that were stressful and/or isolating. To achieve this, the music selected was typically identified as popular, seasonally relevant, and/or aligned with current themes; therefore, it was likely familiar to participants. When less ubiquitous music was introduced, it often shared aesthetic elements with popular music intended to bridge genre divides, prompt conversation, and support social interaction. The workshops were distinguished from clinical music therapy by the absence of individualized symptom assessment and development of specific goals and interventions for each participant. However, this engagement was informed by common music therapy interventions including active music listening, lyric analysis, and songwriting.

**Figure 1:**
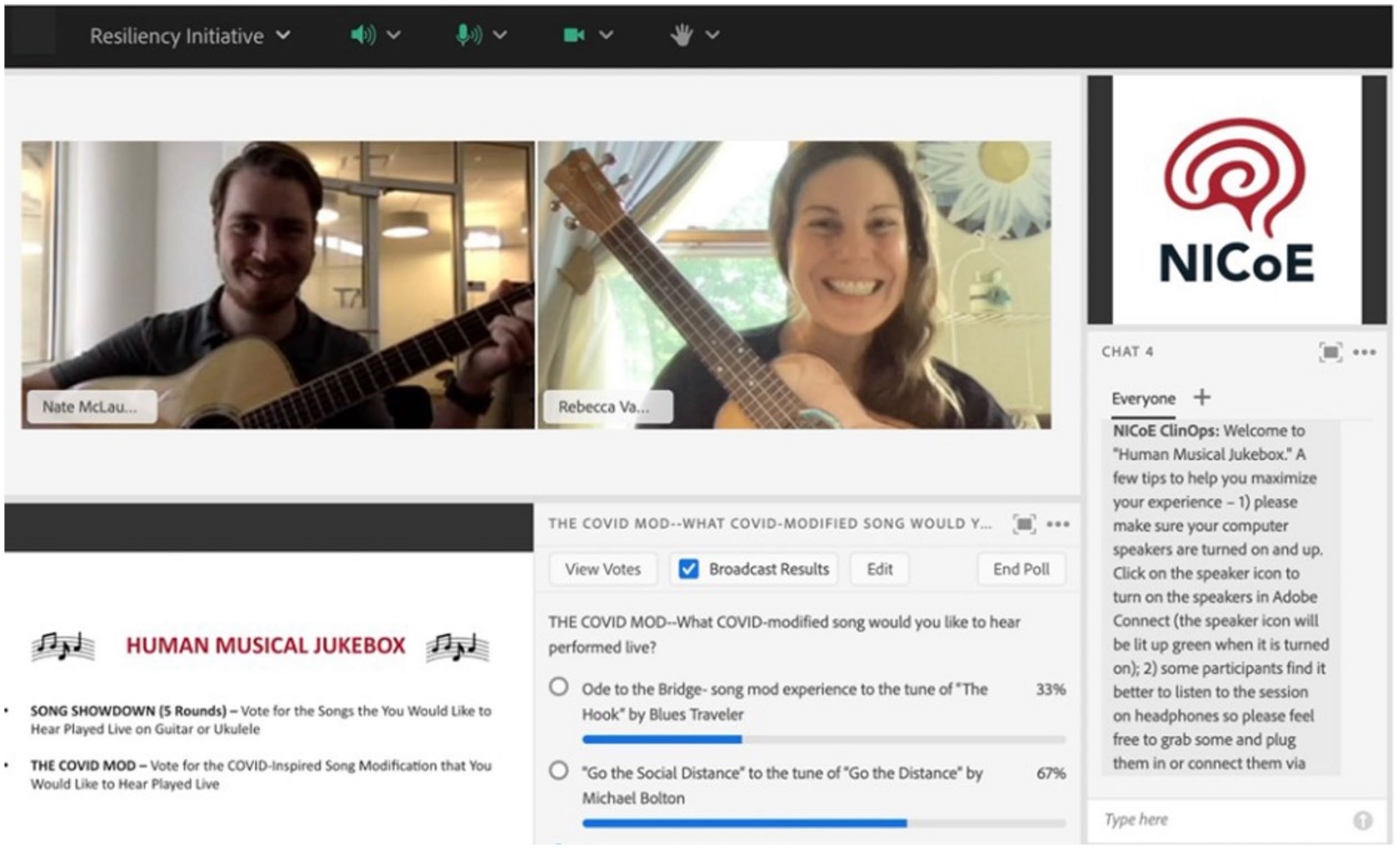
HJB virtual room.

Each week, the music therapists prepared a custom list of songs to perform during HJB, which were pre-populated into the polling function of the virtual room. Songs prepared were selected based on their representation of seasonal themes, chat feedback in prior HJB sessions, potential for inducing mood shifts, and/or reproducibility of musical elements. Workshops began with the music therapists welcoming participants, describing the HJB structure, answering any questions about the process, and directing attention to the poll. Participants selected between two song options that they wanted to hear, resulting in a majority vote for one song. The music therapist who prepared that song would activate their microphone and camera to perform it live using their voice and an instrument of choice for musical accompaniment. Figure 1 demonstrates the HJB workshop virtual room.

Participants voluntarily commented and interacted with one another via the chat throughout the performances. After each song, the music therapists responded to the audience commentary (verbally or via the chat) before initiating the next round of song choices. This promoted an informal dialogue between the facilitators and the participants who chose to interact, as well as amongst the participants communicating with one another. To further promote engagement, the music therapists encouraged participants to request songs for future HJB workshops. The mechanisms underpinning HJB are compliant with the Reporting Guidelines for Music-Based Interventions (Robb et al., 2025). These guidelines were not yet published during the implementation of HJB; however, it is validating that many elements of HJB conceptualization, implementation, and facilitation align with music-based intervention recommendations.

## 4 Human Jukebox thematic analysis methods

Ethics approval: This study was reviewed by the Walter Reed National Military Medical Center Institutional Review Board and was designated as non-research and exempt from ethics committee review. The authors have no competing interests.

Literature indicates that analyzing participant feedback is helpful to identify themes and common experiences that can inform process improvement and provide preliminary insights about participant outcomes (Fraser et al., 2021). Members of the author team, including the two music therapist facilitators, a dance/movement therapist, a doctoral-level program evaluator, and an arts-based community engagement expert, completed a thematic analysis (Braun and Clarke, 2006) of 404 messages that participants posted in the HJB workshop chat from June 2020 to June 2022. The music therapist facilitators compiled the de-identified chat content from each offering in a shared document. Notably, participants entered a username each time they logged into the virtual room, which was the only probable personally identifiable information, and usernames were not included in the shared document.

The thematic analysis was conducted as follows:

1. Each project team member reviewed the chat messages independently and developed unique thematic code words assigned to each message.
2. Project team members met to discuss their unique codes and created a consolidated list of 14 codes based on those that were most commonly applied (Table 2).
3. The program evaluator created operational definitions for each code and met with the project team to review definitions (Table 2).
4. Each project team member coded the messages independently for a second time using the new consolidated code list; team members could use multiple codes for each message.
5. The project team met to discuss differences in their respective coding thought processes to reach a consensus on a most prevalent primary code (Table 3) for each message, and a secondary code (Table 4) for messages by the majority as containing more than one theme.

**Table 2:** Final codes and operational definitions.

Codes	Operational definitions	Example messages
Gratitude	Messages expressing thanks and gratitude for HJB and/or the facilitators	“Thank you for the creativity and the light and joy that you have brought to everyone over the last 2 years!”
		“Bravo! Thank you both for sharing your gift! Much needed! You guys Rock!”
Social connectedness/community engagement	Any messages discussing social connection, shared experiences, the HJB community, including well wishes to other participants and/or facilitators; includes chat conversations between participants and song requests to facilitators	“Good morning from another anywhere/everywhere space”
		“TEAM: Together Everyone Achieves More”
Music appreciation/quality of music/aesthetic appreciation	Messages expressing appreciation for the music being played; included the quality and/or beauty of the performance/song /rendition	“What wonderful song selections to choose from today and thank you for sharing the music video link. Song was amazing and beautiful imagery!”
		“I think the world will be better with that covid-mod”
Informative/educational	Messages that include comments about HJB providing resources, education, information	“Such an educational session today”
		“Learning so much today”
Filling a gap/unique program	Messages about how Operation Oasis is a novel addition and/or how it is meeting a need by providing a program specifically for providers	“This is the first program I’ve seen that focuses on care providers”
		“This should be widely broadcast”
Mindfulness/reflective	Messages indicating participants were focused on the present moment (mindfulness) and/or thinking deeply and/or thoughtfully (reflect); includes comments about spirituality	“Great thoughts ~ discern before reacting = powerful response”
		“It was so nice to reflect. Authentic”
Coping/resilience/helpful/therapeutic	Messages indicating that HJB may have helped participants cope with the demands of life including perceptions of increased resiliency and well-being	“This is motivating me to work harder today for sure or at least not give up when it’s hard!”
		“For me as a Vet … listening to or playing music brings out emotions that have been “put to the side” … now realizing (after a long while … and still continue to do) … they are not necessarily bad”
Positivity/fun/enjoyment	Messages that are positive in tone and/or convey joy and fun; includes expressions of pleasant emotion	“I AM SOOOO BLISSFUL RIGHT NOW!!!!”
		“So happy–hopeful–joyful”
Reminiscence/nostalgia/relatable/relating to music	Messages that include comments about the music evoking memories; includes messages where participants note that the song is relatable to their personal experiences	“Great to hear this important song in my early years”
		“Brings back 80s/90s tennis club waiting area memories -oh those memories”
Sadness/grief/loss	Messages that convey sadness, grief, loss, or other expressions of unpleasant deeper emotion	“So many ways to miss someone”
		“I lost my mom during that long December, need resiliency more than ever!”
Dancing/moving the body/physical reaction	Messages where participants comment that the song is making them dance, move, or have another physical reaction (e.g., clapping along)	“Do not mind me, just dancing around my apartment”
		“Clapping and singing along over here!!!”
Desire to create music/desire of musical skills	Messages where participants express a desire to gain musical skills, play music, and/or create music including writing, singing, or playing a song	“Driving in the car with music is one of my favorite things to do, and singing along!”
		“I wish I could play the ukulele.”
Military service/military pride	Messages mentioning military service and/or having pride for military/veterans/military service	“Sergeant Major (USMC) John Phillip Sousa^a^ would be proud of this rendering, OOOORah!”
		“Veteran using music/creative poetry as tools to manage post-military career”
Relevant to the present time	Messages that include comments about how the song/music being relevant to the current time (e.g., COVID-19, social/political climate, holidays)	“We all need to do this, now especially!!”
		“Perfect song for our current stay at home orders…you guys rock!”

a John Phillip Sousa (1854–1932) was an American bandmaster and composer of military marches, who had enlisted in the U.S. Marine Corps in 1868. Britannica, T. Editors of Encyclopedia (2024, October 4). John Philip Sousa. Encyclopedia Britannica. https://www.britannica.com/biography/John-Philip-Sousa

**Table 3:** Primary code frequencies and percentages.

Code	Frequency	Percentage
Music appreciation	174	43%
Gratitude	47	12%
Positivity	39	10%
Reminiscence/nostalgia/relating to music	25	6%
Social connectedness	25	6%
Mindfulness/reflective	20	5%
Coping/therapeutic	19	5%
Relevant to present time	13	3%
No code	11	3%
Desire to create music/gain musical skills	10	2%
Dancing/moving body	7	2%
Informative/educational	7	2%
Filling a gap/unique program	4	1%
Military service/military pride	2	<1%
Sadness/grief	1	<1%

**Table 4:** Secondary code frequencies and percentages.

Code	Frequency	Percentage
No code	220	54%
Positivity	50	12%
Music appreciation	39	10%
Social connectedness	25	6%
Coping/therapeutic	17	4%
Reminiscence/nostalgia/relating to music	15	4%
Mindfulness/reflective	13	3%
Gratitude	6	1%
Relevant to present time	5	1%
Dancing/moving body	4	1%
Informative/educational	4	1%
Desire to create music/musical skills	2	<1%
Filling a gap	2	<1%
Sadness/grief	2	<1%

### 4.1 Thematic analysis results

Tables 3, 4 display the frequencies and percentages for the primary and secondary codes. They show that 3% of messages did not receive a primary or secondary code (designated as “no code”), and 54% of messages did not receive a secondary code. Music appreciation, gratitude, and positivity were the most common primary codes with 43, 12, and 10%, respectively. Positivity, music appreciation, and social connectedness were the most common secondary codes. Codes that comprised less than 1% of the total message pool were military service/pride, sadness/grief, desire to create music, and filling a gap.

The analysis of HJB chat messages revealed that participants commonly expressed appreciation for the music, gratitude, positivity, feelings of reminiscence/nostalgia relating to music, and feelings of social connectedness during the sessions. These preliminary insights are consistent with several CF community outcomes, specifically creative expression, social connectedness, and resilience. These most common codes also accurately reflected the intended scope and impact of HJB; nearly half the comments referred directly to the music experience.

The least common codes (<1%) appear to be associated with messages of context-significant subject matter, despite being brought up infrequently. Military service and sadness/grief may have been implicit subtext in social situations which were designed around uplifting and connecting through music. Participants who shared those comments may have occasionally felt empowered to share their thoughts in such a supportive environment. They may have responded to specific song themes, instruments, and/or lyrics, though this possible relationship was not measured. Although HJB was facilitated by trained music therapists, the scope was non-clinical and focused on resilience-building and peer connections through music. This shared understanding likely steered participants away from disclosing sensitive personal information.

## 5 Discussion

Operation Oasis, specifically HJB, aimed to support community-building in a non-physical social space by offering weekly opportunities in which providers working in military healthcare could gather and engage in community building through participant-informed live-streamed music performances. These efforts showed scalability potential, as networked providers in the military healthcare system from across the U.S. joined in the effort to facilitate workshops that would accommodate various time zones. This institutional-level investment accommodated participation of individuals who otherwise would not have the ability to participate; hence, promoting social connection in support of the health and safety of the medical community.

Regarding the identified themes, the clear prevalence of music appreciation may be self-evident due to the music-centric nature of the HJB format. However, it also connects meaningfully to the “Music Has No Borders” study, which found that, “acknowledgment of the shared experience of adversity, positive collective responses, and interaction between audience participants represented displays of community resilience” (Fraser et al., 2021, p. 15). Researchers further underpin this concept with the theory that music engagement in the context of limited social availability appears to embody a social surrogate role (Schäfer et al., 2020). The high volume of explicit music appreciation feedback in this study may therefore functionally represent implicit endorsement of social connection. This relationship should be studied further.

The intersections of communications technology and social connection provided through Operation Oasis are reinforced by a report published by the U.S. Surgeon General’s Advisory on the healing effects of social connection and community, which focuses on the effects of loneliness and isolation and outlines the importance of strengthening social infrastructure in communities (Office of the Surgeon General, 2023). The report identifies conceptual pillars to uphold and advance social connection. The intention of the first pillar, which recommends to “design the built environment to promote social connection” and “establish and scale community connection programs,” (2023, p. 48) aligns with the goals of the Operation Oasis.

The report also highlighted how technology can either help or hinder social connection depending on its design and usage, and that using pre-existing virtual environment systems may be time-efficient but may not be optimally impactful. On the other hand, introducing a new virtual platform necessities training to ensure appropriate use and planning for optimal implementation, which requires resources (e.g., time, money) that would not have accommodated the quick pivot that the COVID-19 pandemic demanded. Lastly, the report stressed that safety protocols and design safeguards in these contexts should be frequently updated and continually improved based on the unique variables of different virtual platforms and their user base. (Office of the Surgeon General, 2023). To prioritize safety standards aimed at harm reduction in digital environments, each Operation Oasis workshop began with facilitators advising participants on how to ensure their well-being while engaging. For example, facilitators advised participants to turn down the volume on their devices if they experienced sensory overload, or to leave the virtual room for a break if needed. HJB participants did not experience any known deleterious health effects from engagement.

Further safety considerations underscore rationale for why it was beneficial and arguably necessary that HJB facilitators were music therapists maintaining the Music Therapist – Board Certified credential. Although the workshops were not designed as clinical treatment, music therapists are held to competency standards that include training in anticipating and understanding the potential harms of music-based experiences (Certification Board for Music Therapists, 2019). The facilitators were aware of the potential for music to broach uncomfortable topics, initiate sensory overload, and trigger emotional responses of music within a broad pool of anonymous participants. The facilitators’ past experiences with music-based interventions positioned them to hold space for addressing thoughts and feelings shared by participants in an ethical manner and to reframe dialogue, thus preventing HJB workshops being misconstrued as therapy.

### 5.1 Lessons learned

During the process of developing arts-based Operation Oasis content, music therapists identified effective methods for virtual delivery of these experiences, which they continued to cultivate and disseminate through webinars, presentations, and consultation. One primary challenge of virtual clinical facilitation for healthcare providers was adapting treatment, interventions, and practice methods from in-person clinic spaces to digital platforms. At the rise of the COVID-19 pandemic, formal training regarding this extent of practice adaptation and platform-specific operational guidance was not commonplace in academia nor healthcare systems as institutions were rapidly pivoting and relying on in-the-moment learning (Meese et al., 2022; Trenerry et al., 2021). The NICoE Information Technology team responded by providing foundational trainings to staff on the general capabilities of their approved virtual platform; however, the clinical realities of effectively utilizing audio/video features and optimizing participant interconnectedness required providers to deploy creative problem solving and iteration from individual perspectives.

Prior to the lockdown period, increasing demand for physical treatment spaces at the NICoE created challenges for providers to accommodate scheduling patients for a given service. This has been a known issue for interdisciplinary healthcare institutions (Kim et al., 2020), which was exacerbated by the COVID-19 pandemic (Javed et al., 2022). A secondary benefit of improving staff competency in using virtual spaces was how it alleviated clinical space limitations. In the time since the public health emergency has waned, providers at NICoE have continued to explore ways to expand telehealth capabilities that reduce barriers to care.

Technical takeaways from HJB informed other Operation Oasis offerings, as they all used the same approved virtual platform. These adjustments also contributed to the structure of the offering. For example, simultaneous use of audio between participants and facilitators during synchronous discussion was problematic and unpleasant due to feedback generated between the audio input/output of their respective devices. This quickly prompted a procedural shift of disabling/muting participant microphones and offering the opportunity to contribute feedback using the chat/text feature, which was effective for facilitating live discussion.

In addition to audio challenges, data bandwidth limits often led to latency during concurrent video streams. To compensate during HJB, participants’ cameras were deactivated, and the facilitators took turns using video and audio. For example, when one facilitator was performing, the other would turn off their camera and mute their microphone. A secondary benefit that emerged from these technological restraints was the choice of participants to engage anonymously. In addition to their cameras and microphones being muted, they could choose any username (given name, initials, or pseudonym) upon entering the room. This may have preserved their perception of privacy and therefore safety to express themselves authentically.

Communication and outreach were additional areas of challenge and learning. Because Operation Oasis began uniformly as offerings for facility staff and providers, and only some offerings later shifted towards including patients and family members, and navigating the communication of those changes was inconsistent and caused confusion. Each facilitator wanted to ensure they considered the ethical implications of patient/family inclusion and determine if/how they needed to modify workshop content. Recruitment strategies evolved over time and included QR codes posted on flyers throughout the hospital, weekly email distribution of the schedule, and word of mouth. Inconsistencies in communication may have contributed to variance in numbers of participants, for example, schedules being sent late, from different e-mail addresses, or containing incomplete information. The most likely reason for these inconsistencies was that the personnel responsible for outreach would change periodically, introducing the possibility for variance in task understanding and execution.

Despite technical challenges, an important and overarching lesson learned was that Operation Oasis offerings, including HJB, aimed to support healthcare workers in high-stress environments by offering strategies that may fortify resilience and mitigate burnout during a time of profound uncertainty, anxiety, and rapid change. This aligns with reported outcomes and recommendations from studies on resilience and burnout of healthcare staff during the pandemic. Healthcare workers with high resilience report lower burnout, which reduces absenteeism and presenteeism (Bellicoso et al., 2026). At an organizational-level, leadership across healthcare sectors should consider designing resilience-building efforts to be preventative instead of reactive, which may more effectively impact patient care and hospital operations at-large.

### 5.2 Limitations and future directions

Due to the nature of thematic analysis, this project has limitations compared with a prospective exploratory design. For instance, the true number of participants is unknown due to the variance in participation from week to week and the lack of a formal recruitment process. The coding process itself likely contains inherent biases that could not be fully accounted for or eliminated. The two facilitators of HJB contributed their own theme-message pairings in addition to themes submitted by the other authors (non-facilitators). Additionally, only descriptive statistics were employed, primarily due to the qualitative nature of the data captured, but there may be other findings possible with more data and a deeper statistical analysis.

A future study of a similar program could investigate connections between themes, such as tracking how often a primary theme was correlated with a secondary theme. Furthermore, a more comprehensive qualitative study could be conducted that includes focus groups and/or key informant interviews with participants and facilitators. No demographic or quantitative outcome measure data were collected on participants, which in a future study could provide deeper insights into value and impacts of other programs with related intent and scope.

More research is needed to determine if participants’ perceived resiliency and feelings of social connectedness persisted following participation in the virtual workshops, and if so, for how long. Future studies could explore whether certain subgroups (e.g., type of healthcare provider, clinical practice, prior experience with virtual engagement) are more likely to benefit from participation. If new virtual programming options are developed, it will be useful to learn more about which arts-based modalities are most effective. Additionally, strategic monitoring, evaluation, and other learning efforts are needed, such as developing theories of change, logic models, and formative evaluations specifically measuring impacts and outcomes of virtual workshops.

The Defense Health Agency may have the opportunity to spearhead the research, creation, and evaluation of new social connection and resilience programs, including virtual opportunities, by developing a policy-driven strategy to address social connection and resilience. The strategy should incorporate perspectives of patients, healthcare providers, and healthcare support staff. The Defense Health Agency has publicly stated on its website that “it is essential for overall health and wellness to develop and maintain social connections,” (Military Health System, 2023, para. 6), which demonstrates their awareness of social disconnection as a public health issue. This is also in alignment with the second pillar from the Surgeon General’s report, which states: “…prioritizing social connection in policy agendas and leveraging ‘Connection-in-All-Policies’ approach requires establishing cross-departmental leadership to develop and oversee an overarching social connection strategy” (Office of the Surgeon General, 2023, p. 49).

## 6 Conclusion

Provider response to the COVID-19 global pandemic was not without challenge and placed a nationwide focus on the needs of healthcare workers. Engagement in innovative virtual programming like Operation Oasis created opportunities for providers to develop supportive content for peers and gain critical facilitation experience using virtual platforms that informed clinical telehealth, which remains a viable and popular treatment option that optimizes patient access to healthcare. This article described the conceptualization, development, and implementation of an initiative that integrated arts engagement as a key component of interactive virtual workshops for military healthcare providers during the COVID-19 pandemic. There are many ways to implement this type of supportive programming, and with the experience gained from responding to a period of high tension and unpredictability, future designs of community-oriented arts-based programs have an opportunity increase their breadth and depth of impact.

## Data Availability

Due to regulatory policy, the dataset for the thematic cannot be made publicly available. Inquiries regarding the dataset should be directed to the corresponding author.

## Author disclaimer

The opinions contained herein represent the private views of the authors and are not to be construed as official or as reflecting the views, opinions, or policies of the Henry M. Jackson Foundation for the Advancement of Military Medicine, Inc., the National Endowment for the Arts, Department of War, or the U.S. Government. Mention of trade names, commercial products, or organizations does not imply endorsement by the U.S. Government. This material was created free of branding or market affiliations. The authors are operating solely as contributors.
